# Staged Mucosal Advancement Flap versus Staged Fibrin Sealant in the Treatment of Complex Perianal Fistulas

**DOI:** 10.1155/2011/186350

**Published:** 2011-07-26

**Authors:** S. J. van der Hagen, C. G. Baeten, P. B. Soeters, W. G. van Gemert

**Affiliations:** Department of Surgery, Academic Hospital of Maastricht, 6202-AZ Maastricht, The Netherlands

## Abstract

*Background*. In this prospective randomised study, the staged mucosal advancement flap is compared with staged fibrin sealant application in the treatment of perianal fistulas. *Methods*. All patients with high complex cryptoglandular fistulas were randomised to closure of the internal opening by a mucosal advancement flap (MF) or injection with fibrin sealant (FS) after treatment with setons. Recurrence rate and incontinence disorders were explored. *Results*. The MF group (5 females and 10 males) with a median age of 51 years and a median followup of 52 months. The FS group (4 females and 11 males) with a median age of 45 years and a median followup of 49 months. Three (20%) patients of the MF group had a recurrent fistula compared to 9 (60%) of the FS group (*P* = 0.03). No new continence disorders developed. *Conclusion*. Staged FS injection has a much lower success rate compared to MF.

## 1. Introduction

Complex fistulas are defined as trans-, inter-, extra- and suprasphincteric fistulas with tracts and branching tracts traversing the middle third or upper part of the anal sphincter. For many years, high transsphincteric fistulas were treated by a fistulotomy or a cutting seton. The disadvantage of this technique is that it increases the incidence of continence disorders such as faecal soiling, incontinence for gas or liquid stool. 

At present, closure of the internal opening is the standard procedure in the treatment of high peri-anal fistulas. For this purpose, techniques such as mucosal advancement flaps, plugs, and closure with sealants have been developed [1–4]. Several reports demonstrate rates of recurrence after these techniques between 8% and 40% [2, 4–9]. Especially complex recurrent fistulas with multiple tracks above the middle third of the sphincter are difficult to treat. We therefore adopted a staged strategy, including the use of a noncutting or draining seton in patients with complex fistulas to reduce the associated inflammation before definitive surgical treatment by a mucosal advancement flap [9]. The mucosal advancement flap is a difficult procedure in the treatment of complex high peri-anal fistulas. The recurrence rate is about 20–50%, and despite the fact that the anal sphincter is in principle saved by this technique, continence disorders are still common [2, 8, 10–12]. The procedure requires good surgical exposure for an adequate dissected mucosal flap. Minimal invasive techniques such as fibrin sealant are introduced and described by several authors to achieve a better outcome and prevent continence disorders after treatment [13–17]. Some authors demonstrated fewer recurrences and no complications after repeated injections of fibrin sealant [14, 15, 17]. They suggested that this was due to the fact that this technique is less invasive and leads to less damage of the anal sphincter.

In this study, we compared the rate of recurrence and rate of continence disorders of a staged fibrin sealant therapy with the staged mucosal flap (used as gold standard) for the treatment of complex peri-anal fistulas in a prospective randomised study.

## 2. Patients and Methods

**Figure 1:**
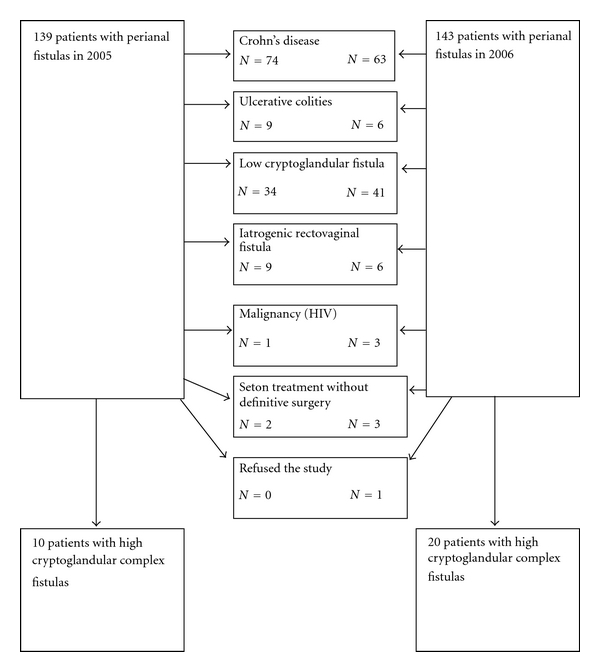
Flow chart of patients with perianal fistulas treated in the Academic Hospital of Maastricht between 2005 and 2006.

Between 2005 and 2006, 30 consecutive patients with complex peri-anal fistula were surgically treated and included in this study (Figure 1). The Academic hospital of Maastricht is a tertiary referral center for patients with complex perianal fistulas. Complex fistulas were defined as transsphincteric, suprasphincteric, and extrasphincteric fistula tracts originating from the middle third or upper part of the anal sphincter. 

The study protocol was approved by the ethical committee. Written informed consent was obtained before the start of the study to continue followup at the outpatient clinic after healing of the fistula. All patients underwent physical examination and laboratory tests. Preoperatively, the patient's complaints of incontinence and soiling were recorded. Definitive categorisation of the fistula was determined by careful examination of the MRI by the surgeon and the radiologist. Patient characteristics, like age, sex, fistula type, and previous surgical attempts, were recorded. Only patients with peri-anal fistulas of cryptoglandular origin were included. Female patients with rectovaginal fistulas were excluded. Patients with Crohn's disease, patients younger than 18, patients with malignancy or HIV were excluded.

### 2.1. Procedure

All patients were examined under general or spinal anesthesia, and setons were placed. The MRI scan was used to find the fistula tracts and their complex branches and abscesses adjacent to and connecting with the fistulae. During seton placement residual abscesses were carefully drained while preserving the surrounding healthy tissues as well as possible, if there was an extra opening (internal or external), they were managed by seton (double seton placement) (Figure 3).

At least 3 months after initial treatment a second examination was carried out under general anaesthesia. After induction of spinal or general anaesthesia, 2200 mg amoxicilline/clavulanate acid was given intravenously. When there was no inflammatory activity at the internal opening of the fistula, 

 the seton was removed and definitive surgery in the form of a mucosal advancement flap (MF) [1, 2] or a fibrin sealant injection (FS) (Figure 4) of the track was performed after curettage with a sharp spoon to remove debris and granulation tissue. 

### 2.2. Randomisation, Stratification, and Intention to Treat

Smoking very likely has a negative influence on the recurrence rate and on outcome [5, 18, 19]. During the second examination under spinal or general anaesthesia, patients who smoked were stratified before randomisation to MF or FS. Randomisation took place by drawing envelopes after a telephone call to the secretary during the second examination before definitive surgery.

Evaluation of the results was carried out according to the intention to treat principle. For instance, fistulotomy had to be performed in case of distal migration of the seton to the lower third of the anal sphincter. When there was still infectious activity around the fistula(s) and drainage of pus after finger compression at both sites, the surgical drainage procedure was repeated by placing (a) new seton(s). These patients were therefore not treated according to the research protocol but were kept in the original randomization and are evaluated as such. In case of persisting infection, a new MRI scan was made after at least 6 weeks before a third look under general anaesthesia was performed.

### 2.3. Examination

Patients were followed at regular intervals of three months at the outpatient clinic and were reexamined for the purpose of this study. The fistula was considered to be healed if there was no drainage of the previous external opening with and without finger compression and when the external fistula orifice(s) was healed and asymptomatic. Special attention was paid to the presence of recurrent fistulas. If there was any doubt regarding the presence of a recurrent fistula tract, an MRI was performed. A “recurrent” fistula, absence of wound healing, and persistent symptoms within 3 months after definitive treatment were defined as a failure of treatment. Continence disorders were scored by the Vaizey incontinence score before treatment and at 6 and 12 months after treatment. Before treatment and at 6 and 12 months after treatment, the patients filled in a KEA quality-of-life questionnaire, a EuroQol-5D instrument which evaluates five health domains: mobility, self-care, usual activities, pain/discomfort, and anxiety/depression in relation to faecal incontinence [20].

### 2.4. Statistical Analysis

A recurrent fistula and incontinence disorder were used as a combined clinical endpoint, the outcome of the KEA quality-of-life score was used as a secondary endpoint. The followup of the patients was stated at least 24 months.

The recurrence rate and percentage of incontinence disorders of treatment by a mucosal flap are 25% and 30%, respectively [5, 8, 9]. Power analysis was based on an expected reduction of 50% of incontinence disorders and the same recurrence outcome in the FS group compared to the MF group. Two groups of 80 patients could have been included in approximately two years.

Statistical analysis included two-tailed T tests, two-way analysis for the observed changes in the faecal incontinence quality-of-life scales. For comparison between the MF group and FS group, chi-square analysis and the Fisher's exact test was used. A probability of 0.05 was considered significant. 

## 3. Results

This study is stopped after the inclusion of 30 patients because of the unacceptable results of the fibrin glue. All patients stayed in followup. After termination of the study, one patient treated with staged fibrin sealant had a recurrent fistula.

### 3.1. Staged Mucosal Advancement Flap

Ten men and 5 women with a median age of 51 years (range 39–70) were treated for complex perianal fistulas by seton drainage followed by a mucosal advancement flap (MF). The median followup after definitive treatment was 52 months (range 26–60). The median interval between seton placement and definitive surgical procedure was 4 months (range 3–15). Six patients were smoking during treatment. The patient characteristics are described in Table 1. Eleven patients (73%) had 2 or more high fistula tracts, and 4 patients (27%) had one high fistula tract detected on MRI. In only 2 patients (13%), an abscess was found on MRI and drained after exploration under general or spinal anaesthesia. In 3 (20%) patients, residual inflammatory activity was seen on MRI (Table 1).

**Figure 2:**
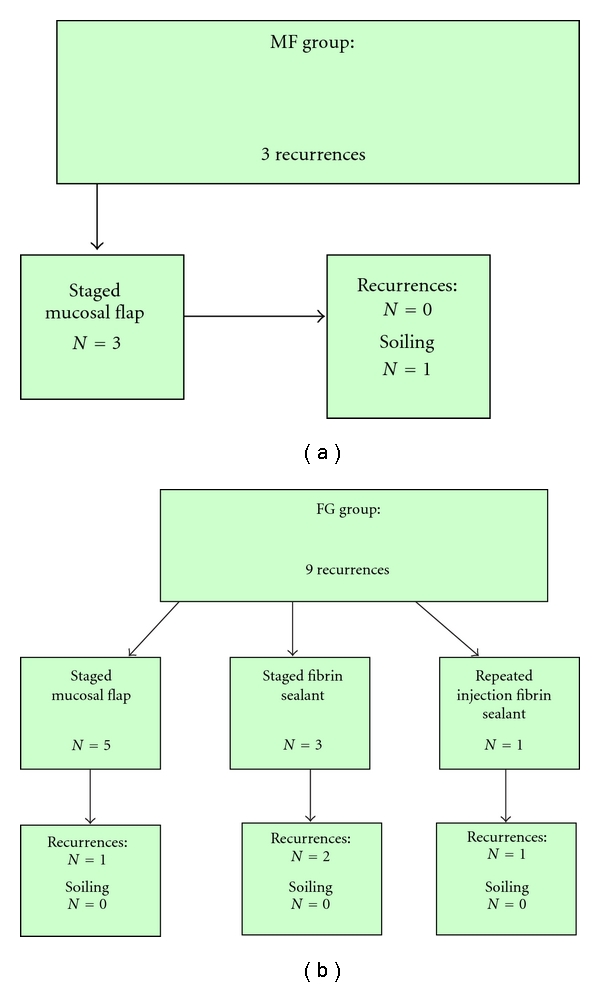
(a) Treatment of patients with recurrent fistulas after MF. (b) Treatment of patients with recurrent fistulas after FS.

**Figure 3:**
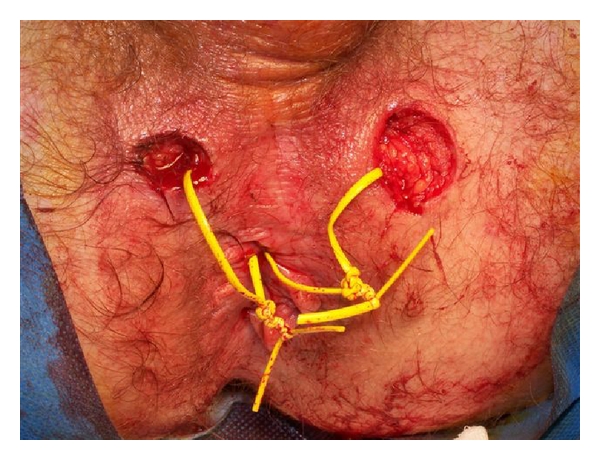
An example of double seton placement in an extra external opening.

**Figure 4:**
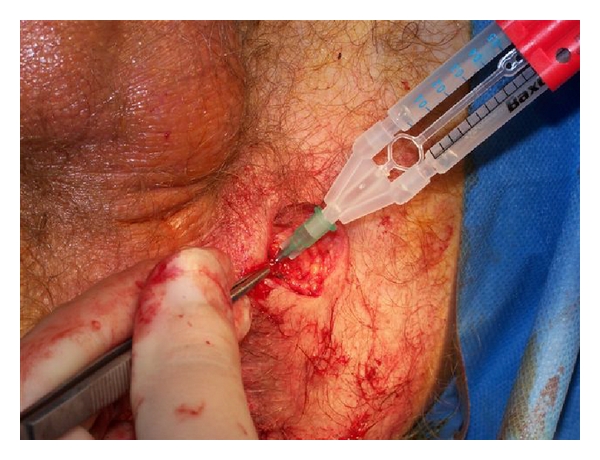
Injection of fibrin sealant injection (FS) after the seton was removed and curetted with a sharp spoon.

 Two patients already suffered from faecal soiling preoperatively, which was unchanged after treatment. No new continence disorders developed after definitive treatment in the other patients. Three (20%) patients developed a recurrent fistula. A new staged mucosal advancement flap was successfully done in all 3 patients without complications (Figure 2).

### 3.2. Staged Fibrin Sealant Treatment

Eleven men and 4 women with a median age of 45 years (range 30–68) were treated for complex peri-anal fistulas by seton drainage followed by fibrin sealant (FS). The median duration of followup after definitive treatment was 49 months (range 29–59). The median interval between seton placement and the definitive surgical procedure was 4 months (range 3–14). Eight patients were smokers during treatment. The patient characteristics are described in Table 1. Ten patients (67%) had two or more high fistula tracts, and five patients (33%) had one high fistula tract seen on MRI. In none of the patients, an abscess was detected on MRI. In 4 (26%) patients residual inflammation was found on MRI (Table 1).

Three patients suffered from faecal soiling preoperatively which remained unchanged after treatment. No new continence disorders developed after definitive treatment in the other patients. Nine (60%) patients developed a recurrent fistula. In 3 patients, a new staged fibrin sealant treatment was performed. In only one patient the repeated treatment was successful. The treatment characteristics are described in Figure 2.

### 3.3. Comparison Between Groups (Table 2)

Three patients (20%) of the MF group and 9 (60%) of the FS group developed a recurrent fistula (*P* = 0.06). Nine (64%) of all the smoking patients (*n* = 14) and 3 (18%) of the no-smoking patients (*n* = 16) had a recurrent fistula (*P* = 0.02).

Eight patients (100%) of the tobacco smokers developed a recurrent fistula in the FS group, and one patient (17%) of the tobacco smokers developed a recurrent fistula in the MF group (*P* < 0.001).

No difference in quality of life (EuroQuol-5D) was found 6 and 12 months after treatment between both groups. Previous surgical attempts, sex, and age were not associated with outcome in both groups.

In this study, no distal migration of the seton, enabling simple fistulotomy, was found.

## 4. Discussion

Low inflammatory activity at the site of the fistula may provide a better chance of definitive cure by ultimate surgical treatment. In a previous study, we have shown that a recurrence rate of 22% can be achieved if the mucosal advancement flap is preceded by at least 3 months seton drainage [9]. The present study shows a less favourable result for staged fibrin sealant treatment. Healing of a high fistula requires adequate closure of the internal opening, which may not be achieved with fibrin sealant. The length of time of adequate sealing of the curetted fistula track by fibrin sealant is probably too short to allow successful formation of a definitive scaffold to close the internal opening. Buchanan et al. could not find remaining traces of fibrin sealant microscopically as early as two weeks after injection [21]. Other studies reported high recurrence rates from 40% up to 86% after treatment with fibrin sealant with or without repeated injections in low as well as in high peri-anal fistulas [14, 17, 22, 23]. Lindsey et al. found a recurrence rate of 31% in complex fistulas and 50% in low fistulas treated with fibrin sealant [13]. But they all reported no continence disorders and described the injection of fibrin sealant as an easy technique [13, 14, 17, 22, 23]. Alexander et al. also found adverse effects, like prolonged severe pain, discharge of great amounts of purulent liquid from the external opening, and abscess formation of fibrin sealant in transanal advancement flap repair [24].

Zimmerman et al. reported that tobacco smokers treated for peri-anal fistulas with transanal advancement flap repair have a 20% higher recurrence rate compared to patients without a smoking habit [5]. They suggested that a nicotine induced decrease in rectal mucosal perfusion contributes to the breakdown of the advancement flap [18]. Our study shows an even greater negative impact of smoking on the success of staged fibrin sealant treatment compared to the staged mucosal advancement flap. This suggests that smoking always affects the outcome of peri-anal fistula repair but especially in inferior treatment modalities. Smoking is also a risk factor for wound healing in perineal wounds [25, 26]. Closure of the internal opening occurs following the general principles of wound healing. A mucosal defect exists which needs to be bridged by influx of white cells, macrophages, fibroblasts, and proteins, all together leading to the formation of a scaffold for further tissue repair, including deposition of collagen, fibrous tissue, and capillarization. It can be assumed that mucosal advancement techniques potentially allow primary wound closure, while fibrin sealant only accelerates scaffold formation, after which secondary wound healing and epithelialisation are necessary. Secondary wound healing requires more of the intrinsic healing potential of the tissue than primary repair. Smoking is known to interfere with many of the stages in wound healing. The synthesis of collagen in smokers is impeded [27]. Nicotine is a vasoconstrictor that reduces the nutritional blood flow to the wound. The proliferation of red blood cells, fibroblasts, and macrophages is reduced by nicotine. Oxygen transport and metabolism is diminished by carbon monoxide and hydrogen cyanide inhibits enzyme systems necessary for the oxidative metabolism and transport at cellular level. The negative influence on wound repair is of particular concern in plastic and reconstructive surgery. Because of the obvious correlation between the number of cigarettes smoked and the healing rate of the wound patients should be advised to quit smoking at least a month before performing elective surgery [28]. 

## 5. Conclusions

Based on the results of the present study, it is concluded that a staged fibrin sealant injection has a lower success rate compared to a staged mucosal advancement flap, especially in patients that smoke. However, after the drawback of this study, the number of patients in both groups is small. But the results of treatment with fibrin sealant after seton placement are unacceptable. Although the staged mucosal advancement flap is a technically more demanding procedure compared to the injection of fibrin sealant, the complication rate is low for both procedures in the hands of dedicated surgeons. In this and another study, a repeated fibrin sealant injection in patients with a recurrent fistula has a lower healing rate and thus requires more procedures compared to a repeated staged mucosal flap [29]. Moreover because fibrin sealant is expensive, a repeated staged mucosal flap is a better choice in patients with recurrent perianal fistulas.

## Figures and Tables

**Table:** (a)

	MF	FS	
	*N* = 15	*N* = 15	
1 fistula track	*N* = 4 (27%)	*N* = 10 (67%)	*P* = 0.15
2 or more fistula tracks	*N* = 11 (73%)	*N* = 5 (33%)	*P* = 0.08
Abscess	*N* = 2 (13%)	*N* = 0 (0%)	*P* = 0.20
Residual inflammation and prolonged seton treatment	*N* = 3 (20%)	*N* = 4 (27%)	*P* = 0.30

**Table:** (b)

	MF	FS	
	*N* = 15	*N* = 15	

Sex	Male *N* = 10 (67%)	Male *N* = 11 (73%)	*P* = 0.55
	Female *N* = 5 (33%)	Female *N* = 4 (27%)	
Age (years)	51 (range 39–70)	45 (range 30–68)	*P* = 0.48
Median followup (months)	52 (46–60) months	49 (49–59) months	*P* = 0.45
Tobacco smokers	*N* = 6 (40%)	*N* = 8 (53%)	*P* = 0.30
Soiling (before treatment)	*N* = 2 (13%)	*N* = 3 (20%)	*P* = 0.40

**Table 2:** Table Patients outcome.

	MF	FS	
	*N* = 15	*N* = 15	
Failure of treatment	*N* = 0	*N* = 0	
Recurrent fistula	*N* = 3 (20%)	*N* = 9 (60%)	*P* = 0.03
Soiling after treatment	*N* = 0	*N* = 0	
Recurrent fistula	(*N* = 6)	(*N* = 8)	*P* = 0.000
In tobacco smokers	*N* = 1 (17%)	*N* = 8 (100%)	
Median quality-of-life score:			
Before treatment	85	87	*P* = 0.32
After 6 months	86	90	*P* = 0.44
After 12 months	87	84	*P* = 0.50
Vaizey incontinence score			
Before treatment	0.50 (0–4)	0.73 (0–4)	*P* = 0.76
After 6 months	0.50 (0–4)	0.73 (0–4)	*P* = 0.76
After 12 months	0.50 (0–4)	0.73 (0–4)	*P* = 0.76
